# Spatiotemporal distribution of microplastics and associations with river water physicochemical and hydrological parameters, including risk assessments in four tropical rivers in Sarawak, Malaysia

**DOI:** 10.1007/s10661-026-15554-0

**Published:** 2026-06-13

**Authors:** Audrey Primus, Tony Hadibarata, Muhammad Noor Hazwan Bin Jusoh

**Affiliations:** Environmental Engineering Program, Department of Civil and Construction Engineering, Curtin University Malaysia, CDT 250, 98009 Miri, Malaysia

**Keywords:** Riverine microplastic, Surface water, Seasonality, River water physicochemical parameters, River hydrological parameters, Risk assessments

## Abstract

The microplastic pollution of freshwater systems has been a global environmental concern, yet there is a lack of knowledge on tropical rivers. This paper examined the relationships between spatiotemporal microplastic abundance and river water physicochemical and hydrological parameters in the surface water of four rivers of Sarawak in Malaysia (Baram, Miri, Sibuti, and Niah), followed by ecological risk assessment using PLI, PHI, and PERI indices. Surface waters were collected during dry and wet seasons and analyzed using stereomicroscopy and ATR-FTIR spectroscopy. The abundance of microplastics ranged from 6.33 ± 1.53 to 27.67 ± 2.31 items/L. Two-way ANOVA revealed a significant spatial difference (*p* < 0.001), but no seasonal difference (*p* > 0.05). The microplastics were predominantly fibers, in black, blue, and clear colors, and 100–1000 µm in size. Dominant microplastic polymers were polyethylene and polypropylene. During the dry season, microplastic abundance correlated positively with salinity (*ρ* = 0.799) and temperature (*ρ* = 0.708) but negatively correlated with pH (*ρ* = −0.764). No significant correlations were found in the wet season. PLI values exceeded 1 in all rivers, while PHI values varied among rivers and seasons (7.16–536.46) (Category II: moderate–IV: danger). The presence of high-hazard polymers, such as rubber, elevated polymer-based risks and increased PERI values in some locations. This study provides baseline knowledge on the seasonal effects and anthropogenic pressures on microplastic distribution in tropical rivers in Sarawak, Malaysia.

**Supplementary Information:**

The online version contains supplementary material available at 10.1007/s10661-026-15554-0.

## Introduction

Plastic production has increased significantly over the past decades, reaching hundreds of millions of tons annually, with a substantial proportion ultimately entering the natural environment (GESAMP, 2019; PlasticsEurope, 2025; Thompson et al., 2004). Due to their durability and resistance to degradation, plastic materials persist and progressively fragment into smaller particles known as microplastics, typically defined as plastic particles 1–5000 µm in size (Frias & Nash, 2019).

Rivers are key pathways in the transportation and redistribution of land-based plastics to the marine environments. The amount of plastic that is transported to the oceans through river water discharge is estimated to range between 1.15 and 2.41 million tons yearly (Lebreton et al., 2017). The amount of ocean plastic on the planet was estimated to grow to 12,000 million tons by 2055 (Chamas et al., 2020). The riverine systems are conduits and temporary sinks that combine urban runoffs, effluents of wastewater, industrial discharge, agricultural activities, and atmospheric deposition (Biao et al., 2025; Curren & Leong, 2023; Hassan et al., 2025; Sekar & Sundaram, 2025).

Although there is growing interest in freshwater microplastics, the comparability of different studies is difficult due to variations in methodology (Nguyen et al., 2022). In Southeast Asia, several studies have reported microplastic contamination, such as in Thailand (Pradit et al., 2023), Vietnam (Le et al., 2026), Indonesia (Ismanto et al., 2023), and Malaysia (Tee et al., 2020), indicating widespread presence of microplastics across the regions. Temporal variability adds another layer of complexity as fluvial systems are highly dynamic. Hydrodynamic and environmental factors (temperature, turbidity, salinity, flow velocity, wind) and particle properties (size, charge, density, composition) affect microplastic abundance (Le et al., 2026). Despite this, there are still limited studies incorporating microplastic abundance with environmental conditions in Southeast Asia.

Malaysia is a significant contributor to the global plastic sector and has reported microplastic pollution in several freshwater systems (Hassan et al., 2025; Tan et al., 2022). Yet, microplastic studies in the state of Sarawak (Choong et al., 2021; Karing et al., 2023; Liong et al., 2021; Mishra et al., 2023), which has major rivers, have remained scarce and only focused on abundance and characterization in water and sediment matrices.

Ecological effects of microplastics depend on particle abundance as well as polymer type, whereby additives and monomers that make up a polymer have the potential to be hazardous to a certain extent. The hazard-ranking framework developed by Lithner et al. (2011) allows for classifying hazards based on the polymer. Polymer load index (PLI) developed by Tomlinson et al. (1980) measures the risk associated with the abundance of pollutants, while the potential ecological risk index (PERI) offers a reference on the potential ecological risk in freshwater systems by combining the previous indices (Baycan et al., 2025; Le et al., 2026).

Thus, this study aims to (i) quantify microplastics in the surface water during dry and wet seasons in four rivers in Sarawak (Baram, Miri, Sibuti, Niah) with varying anthropogenic pressures, (ii) characterize the morphology of the particles and microplastic polymer composition, (iii) examine relationships between microplastic abundance and physicochemical and hydrological parameters, and (iv) undertake a preliminary microplastic risk assessments based on polymer load index (PLI), polymer hazard index (PHI), and potential ecological risk index (PERI). Given the rapid industrialization and urbanization, the distribution of microplastics in tropical river ecosystems, especially in understudied areas such as Sarawak that has major rivers and mangrove ecosystems, is vital for local environmental management and global efforts to tackle plastic pollution and its associated risks.

## Methodology

### Study area and sampling design

**Fig. 1:**
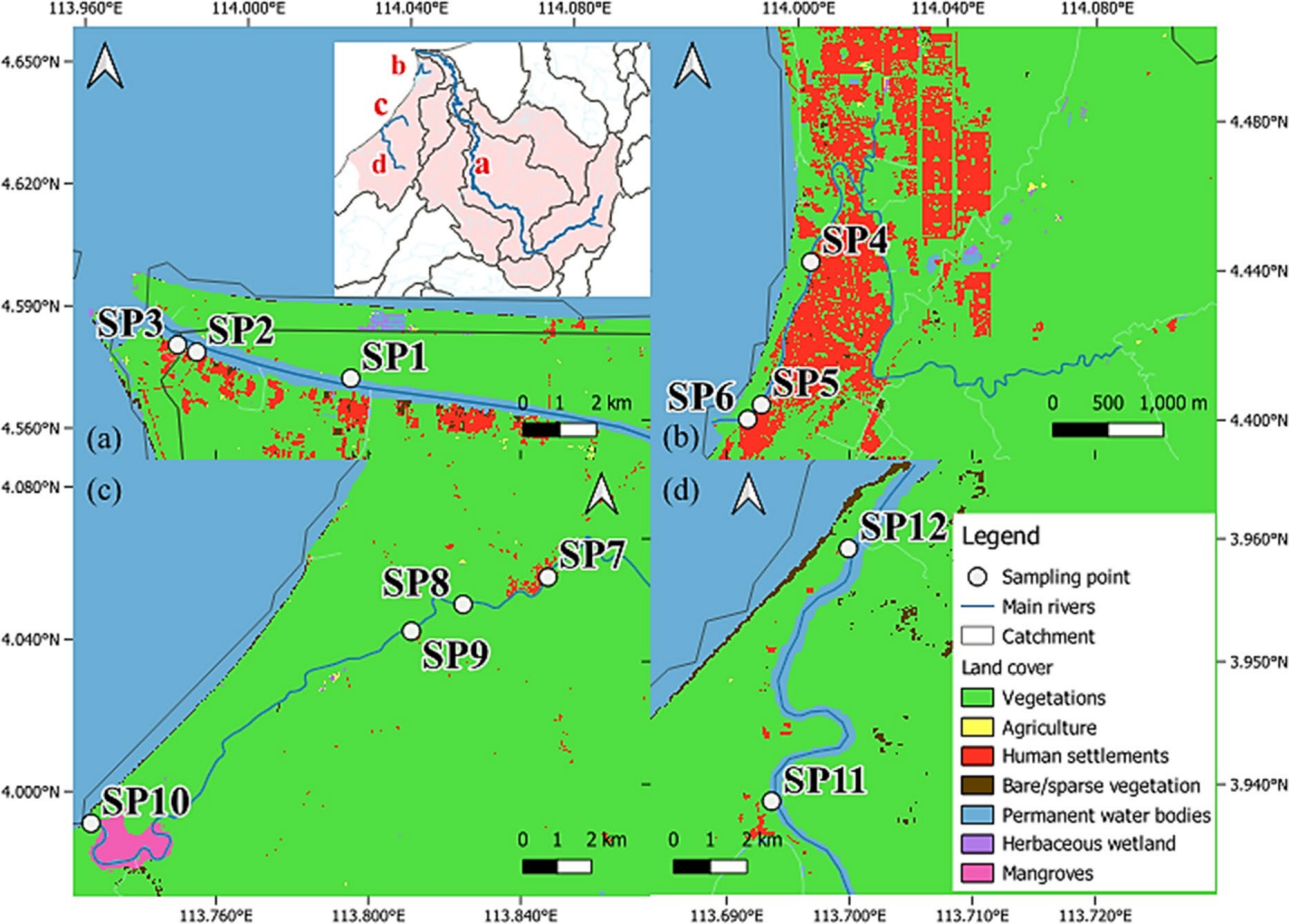
Sampling points of microplastics in the surface water of (**a**) Baram River (SP1–SP3), (**b**) Miri River (SP4–SP6), (**c**) Sibuti River (SP7–SP10), and (**d**) Niah River (SP11–SP12)

The research was carried out in four rivers (Baram, Miri, Sibuti, and Niah) situated within the Miri Division in the Sarawak state of Malaysia. The surface water was sampled at 12 sampling points (Fig. 1). Sampling locations were chosen to reflect various land use types and river features (Table S1). The Baram River, a major river in Sarawak, is approximately 980 km long, with a catchment area spanning approximately 22,800 km^2^ (Choong et al., 2021). The Baram estuary supports various industrial activities such as the plywood industry, sawmill, and shipping and dockyard activities.

The Miri River, which has a length of 45 km and a catchment area of 582 km^2^, drains from Miri Town into the South China Sea (William et al., 2024), and represents a more urbanized area. Both the Sibuti and Niah rivers are characterized by the presence of villages, forested areas, and agricultural lands. The Sibuti River, located on the northwest coast of Sarawak, Malaysia, is 35 km long with a catchment area of 1020 km^2^ and supports mangrove vegetation (Gantayat et al., 2023). Meanwhile, the Niah River, which has a length of 113.12 km and a catchment size of 1349.4 km^2^, provides vital protein sources to the local Iban communities and is also a site for oil palm plantations (University Malaysia Sarawak, 2025).

Sampling was conducted during two seasonal periods, and these periods were defined using short-term rainfall patterns and forecasts characterized by a dry season period (≥ 1 week with no/low rainfall) and a wet season period (≥ 1 week with frequent rainfall events). Sampling was conducted in April and July 2024 (dry season) and January to February 2025 (wet season) in accordance with the Northeast and Southeast monsoons that affect Sarawak (anak Alexander Tampang & Mohan Viswanathan, 2022).

### Surface water sampling

Surface water sampling was conducted once per season without consideration of tidal stage. A pre-cleaned stainless-steel bucket was used to collect 10 L of bulk surface water sample (0–0.20 m depth) from near-bank (riparian) locations. A total of 24 samples (*N* = 24) were collected across four rivers and two seasons, with one sample per sampling point. The samples were transferred into clean containers, transported to the laboratory, and kept in a dark storeroom.

### Microplastic extraction

For each sampling point, the bulk sample (10 L) was thoroughly homogenized by gentle stirring and subsampled into triplicate 1 L aliquots. As the study rivers exhibited high turbidity and suspended sediment load, vacuum filtration was applied using 0.45-µm membrane filters (Labfil Solution) to reduce sample volume and to minimize reagent usage. The contents that remained on the membrane were then rinsed into a 250-mL conical flask using distilled water up to the 150-mL mark.

Organic digestion was performed using Fenton’s oxidation method (Masura et al., 2015), which is widely used to extract microplastics from environmental samples (Sekar & Sundaram, 2025; Sulistyowati et al., 2022). The procedure was modified to remove additional heating, but with an extended digestion time. This step was applied to minimize potential thermal degradation of microplastics, as the reaction itself is exothermic (Munno et al., 2017). Twenty milliliters of 30% hydrogen peroxide (H_2_O_2_, Bendosen) and 0.5 mL of 0.05 M FeSO_4_ catalyst solution (prepared by dissolving 7.5 g FeSO_4_·7H_2_O, Merck, in 500 mL distilled water) were poured into the flasks, sealed with aluminum foil, and left undisturbed for 24 h.

Density separation was performed by adding zinc chloride solution (ZnCl_2_, PC) adjusted to a density of 1.6 g/cm^3^ (approximately 43–45% w/w), prepared similarly to those described by Coppock et al. (2017). The ZnCl_2_ solution was poured up to the 250 mL mark on the conical flask, then shaken briefly, and allowed to settle for 24 h. The supernatant was then vacuum-filtered onto the 0.45-µm filters, placed in pre-cleaned and pre-labelled Petri dishes, and dried overnight in an oven (Memmert UM 500) at 30–40 °C. Petri dishes were sealed with masking tape and stored prior to stereomicroscopy and polymer validation.

### Microplastic identification and validation

After extraction, the abundance and characteristics of microplastics (shape, color, size) were measured using a camera-equipped stereomicroscope (Nikon SMZ745T) at magnifications of 20×–40× and using a zigzag scanning technique to cover the entire surface of the filter. Microplastics were categorized into fiber, film, fragment, pellet, foam, and bead. Meanwhile, colors were categorized into common color groups such as black, blue, green, clear, white, red, and yellow. Although 0.45-µm filters were used for filtration, the effective detection limit was constrained by microplastic size definition (1–5000 µm); therefore, reported size classes were divided into 1–10 µm, 10–100 µm, 100–1000 µm, 1000–2000 µm, and 2000–5000 µm.

A total of 134 particles were selected randomly across different shapes, colors, sizes, sampling locations, and both seasons to ensure representative polymer coverage. Agilent Fourier Transform Infrared Spectroscopy (FTIR) (Agilent, Cary 630, Serial No. MY17172006) with diamond ATR accessory was used to identify the polymers. Spectra were acquired over 650–4000 cm^−1^ at 4 cm^−1^ resolution using MicroLab software, with 32 sample scans and 8 background scans. Validation was done using pre-loaded spectral libraries, and particles that were spectral matched (> 70%) were considered microplastics (Kong et al., 2024). The ATR crystal was cleaned with 2-propanol (Merck) prior to and after each analysis to avoid cross-contamination, and particles were handled using stainless-steel forceps.

### Measurement of river water physicochemical and hydrological parameters

During sampling of surface water, the in situ river water physicochemical parameters (turbidity, temperature, salinity, pH) were measured using a portable turbidimeter (HACH 2100Q, USA), a Pocket Probe Pro tester (pH and temperature), and a salinity meter (Eutech Salt 6) with an electrode ECCONSEN91B. After every measurement, the probes were cleaned with distilled water. To describe the conditions of the rivers and compare them with the abundance of microplastics, river hydrological parameters (river velocity, river depth, water discharge, including rainfall) were also assessed during both seasons (Table S4, S5).

A flowmeter (Aermanda FM-210 V5) was attached to a 1.20-m metal pole and used to measure the velocity of the river at three cross-sectional positions (left bank, mid-channel, right bank) at 10-s intervals in units of velocity (m/s). From the boat, the flowmeter was placed at a depth of nearly 15–40 cm from the water surface to indicate near-surface flow and, where possible, at the lowest depth that could be reached (~ 60–150 cm) to indicate mid-water flow. Due to equipment length and safety constraints in very deep channels, measurements represent point velocities rather than full vertical velocity profiles. Sampling point velocity was obtained by averaging replicate measurements at each cross-sectional position across the river section. The depth of the river was determined by submerging a portable sonar depth checker (LUCKY FF1108-1CWLA) in the water (on a fishline), and the width of the river was measured using a rangefinder (MILESEEY-PF260) across opposite banks.

Following the approach described by Chen et al. (2022) and Pertiwi et al. (2025), the river water discharge (m^3^/s) was calculated using the velocity-area method and trapezoidal approximation using Eq. (1):

1 $${q}_{\mathrm{i}}={\overline{u} }_{\mathrm{i}}\left(\frac{{b}_{i-1}+ {b}_{i+1}}{2}\right){d}_{\mathrm{i}}$$

*q*_i_ is the discharge for subsection *i* (m^3^/s); *u*_i_ is the mean velocity at subsection *i*, which was calculated as the average of the surface velocity and middle-depth velocity at that location; *b*_i-1_ + *b*_i+1_ are the widths of the adjacent subsections on either side of the current subsection, which are averaged to account for changes in the river width; and *d*_i_ is the depth of subsection *i*, measured at the center of the subsection. The total discharge was obtained by adding the representative discharges.

One-week hourly antecedent rainfall readings (mm) and 1-week hourly antecedent water level relative to flood gauge readings (m) before sampling were obtained from the Department of Irrigation and Drainage (DID) Sarawak in Malaysia through a formal request for academic use. DID gauging station records of hourly rainfall and water level were extracted and averaged over a constant 7-day period before the last sampling time of each river sampling event. River-level seasonal means ± SD were computed for river water physicochemical and hydrological parameters.

### Quality assurance and quality control (QA/QC)

All solutions involved in the laboratory processing were pre-filtered before use in order to reduce contamination. A damp filter paper was placed near the work area during procedures that were done outside the fume hood to monitor airborne contamination. In this study, however, no airborne microplastics were observed. Laboratory coat and nitrile gloves were worn throughout processing, and any solution or sample was covered whenever possible. A previous study has applied a similar QA/QC procedure in addition to conducting procedural blanks (Valsan et al., 2024). Therefore, procedural blanks should be considered in future studies as a way of increasing data reliability.

### Statistical analysis

The abundance of microplastics was given as items/liter and was summarized as the mean and standard deviation (SD) of the three 1-L subsamples by sampling points and season. The distribution of shapes, colors, sizes, and polymers of microplastics was visualized using 100% stacked bars. Two-way ANOVA with Tukey’s post hoc test was used to test the difference in abundance between rivers and between seasons. The Shapiro-Wilk test was used to check the normality of the data. Subsequently, the abundance of microplastics was correlated with river water physicochemical and hydrological parameters with Spearman’s rank correlation, with a significance level of *p* = 0.05. A total of 24 samples (12 sampling points across four rivers and two seasons) were included in the analysis. All statistical analysis and graphing were conducted in Origin 2024 software.

### Preliminary ecological risk assessments

#### Pollution load index (PLI)

The pollution load index (PLI) has been extensively used to study the content of estuarine contaminants (Tomlinson et al., 1980). It has since expanded to investigate the status of microplastic pollution (Chen et al., 2026; Le et al., 2026; Wang et al., 2021). To estimate the PLI value in Eq. (3), the contamination factor (CF_i_) was computed using Eq. (2) (Tomlinson et al., 1980) while the PLI score was determined by using Eq. (3) (Chen et al., 2026; Le et al., 2026; Sekar & Sundaram, 2025).

2 $${\mathrm{C}\mathrm{F}}_{\mathrm{i}}=\frac{{C}_{\mathrm{i}}}{{C}_{0}}$$

3 $$\mathrm{P}\mathrm{L}\mathrm{I}= \sqrt{{\mathrm{C}\mathrm{F}}_{\mathrm{i}}}$$

4 $${\mathrm{P}\mathrm{L}\mathrm{I}}_{\mathrm{R}\mathrm{i}\mathrm{v}\mathrm{e}\mathrm{r}}= \sqrt[\mathrm{n}]{{\mathrm{P}\mathrm{L}\mathrm{I}}_{1}\times {\mathrm{P}\mathrm{L}\mathrm{I}}_{2}\times {\mathrm{P}\mathrm{L}\mathrm{I}}_{3}\dots \times {\mathrm{P}\mathrm{L}\mathrm{I}}_{\mathrm{n}}}$$

The microplastic abundance factor (CF_i_) is the ratio between the microplastic concentration *C*_i_ and the baseline of microplastic concentration *C*_0_ that would not cause adverse effects on aquatic organisms, which was estimated to be 6.65 items/L based on the meta-analysis of the existing literature (Chen et al., 2026; Everaert et al., 2018). The PLI represents the pollution load index of a single sampling point, and PLI_River_ represents the pollution load index for the river (Eq. 4), where *n* is the number of sampling points (Le et al., 2026; Wang et al., 2021). A PLI value of > 1 indicates a significant presence of pollution at the site (Chen et al., 2026).

#### Pollution hazard index (PHI)

Polymeric hazard risk of microplastic contamination was estimated based on the chemical composition of microplastics and hazard scores that were established by Lithner et al. (2011) as the chemical toxicity coefficient of the identified microplastic polymers. To determine the ecological hazard of microplastic contamination, the polymer hazard index (PHI) of a sampling point in Eq. (5) was used (Le et al., 2026; Wang et al., 2021).

5 $$\mathrm{P}\mathrm{H}\mathrm{I}=\sum \left({P}_{\mathrm{x},\mathrm{i}}\times {S}_{\mathrm{x},\mathrm{i}}\right)$$

6 $${\mathrm{P}\mathrm{H}\mathrm{I}}_{\mathrm{R}\mathrm{i}\mathrm{v}\mathrm{e}\mathrm{r}}= \sqrt[n]{{\mathrm{P}\mathrm{H}\mathrm{I}}_{1}\times {\mathrm{P}\mathrm{H}\mathrm{I}}_{2}\times {\mathrm{P}\mathrm{H}\mathrm{I}}_{3}\dots \times {\mathrm{P}\mathrm{H}\mathrm{I}}_{n}}$$

*P*_x__,__i_ is the percentage of microplastic polymer *x* identified at sampling point *i* using ATR-FTIR and *S*_x,i_ is the hazard score of a single polymer *x* (PP = 1, PE = 11, PET = 4, PS = 30, Rubber = 1628), following Lithner et al. (2011) and subsequent studies (Baycan et al., 2025; Le et al., 2026; Wang et al., 2021; Ziembowicz & Kida, 2025).

PHI_River_ in Eq. (6) refers to the risk index of a river, where *n* is the total number of sampling points. Microplastic polymers in low quantities, indicated as “Others,” were ignored, and percentages were not renormalized. PHI was determined at sampling points where data on polymer composition were available due to the limitations of ATR-FTIR. Based on the PHI values, the level of ecotoxicological risks can be assessed, namely Category I (minor, 0–1), Category II (moderate, 1–10), Category III (high, 10–100), Category IV (danger, 100–1000), and Category V (extreme danger, > 1000) (Baycan et al., 2025; Lithner et al., 2011; Ziembowicz & Kida, 2025).

#### Potential ecological risk index (PERI)

The potential ecological risk index (PERI) of microplastics takes into consideration the impact of the abundance of microplastics, as well as the impact of the types of polymers, on the ecological risk posed by microplastics (Le et al., 2026; Wang et al., 2021). PERI_i_ at a sampling point *i* was determined by using the following Eq. (7), and PERI_River_ of a river can be calculated using Eq. (8) where *n* is the number of sampling points.

7 $${\mathrm{P}\mathrm{E}\mathrm{R}\mathrm{I}}_{\mathrm{i}}= {\mathrm{C}\mathrm{F}}_{\mathrm{i}}\times {\mathrm{P}\mathrm{H}\mathrm{I}}_{\mathrm{i}}$$

8 $${\mathrm{P}\mathrm{E}\mathrm{R}\mathrm{I}}_{\mathrm{R}\mathrm{i}\mathrm{v}\mathrm{e}\mathrm{r}}= \sqrt[n]{{\mathrm{P}\mathrm{E}\mathrm{R}\mathrm{I}}_{1}\times {\mathrm{P}\mathrm{E}\mathrm{R}\mathrm{I}}_{2}\times {\mathrm{P}\mathrm{E}\mathrm{R}\mathrm{I}}_{3}\dots \times {\mathrm{P}\mathrm{E}\mathrm{R}\mathrm{I}}_{n}}$$

The PERI was classified as low for values < 150, moderate for 150–300, high for 300–600, danger for 600–1200, and extreme danger for values > 1200 (Le et al., 2026; Sekar & Sundaram, 2025).

## Results

### Spatiotemporal abundance of microplastics

**Fig. 2:**
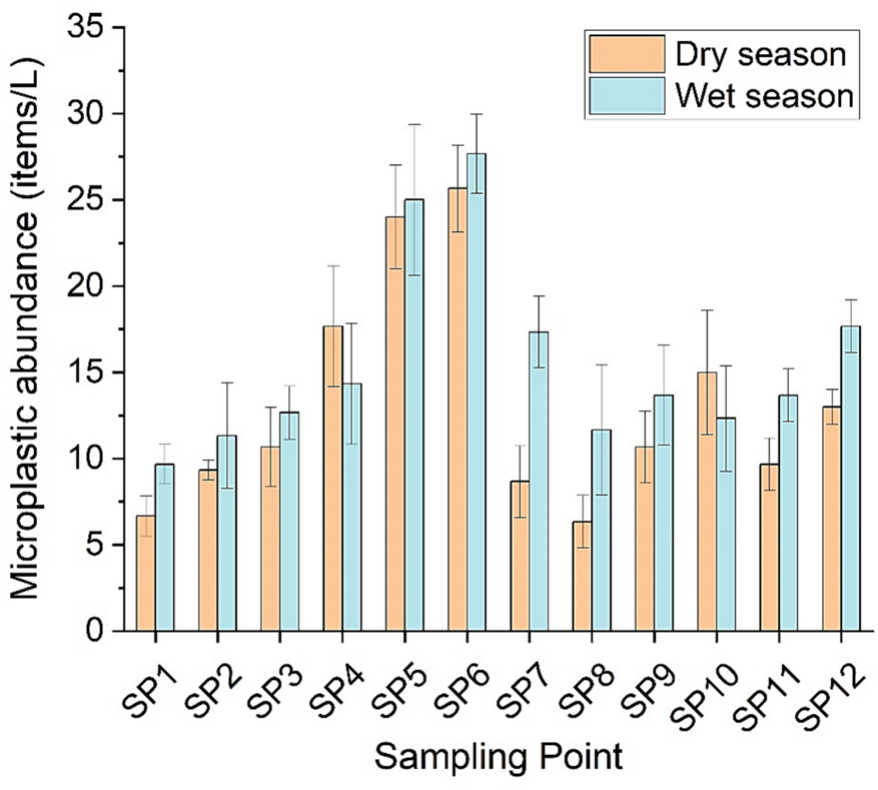
Seasonal microplastic abundance at sampling points (SP1–SP12) in the Baram, Miri, Sibuti, and Niah rivers

The mean abundance of microplastics was different among rivers and seasons, varying between 8.89 ± 1.35 and 22.44 ± 3.01 items/L. Microplastic abundance in the surface water during the wet season was higher than during the dry season for most sampling points (Fig. 2). The Miri River showed the highest mean abundance during the dry season (22.44 ± 3.01 items/L) and the wet season (22.33 ± 3.39 items/L). In contrast, the Baram River showed lower values (dry: 8.89 ± 1.35 items/L, wet: 11.22 ± 1.91 items/L), while the Sibuti River exhibited intermediate mean microplastic abundance (dry: 10.17 ± 2.32 items/L; wet: 13.75 ± 2.95 items/L). The Niah River had slightly higher values than Sibuti, with mean abundance values of 11.33 ± 1.26 in the dry season and 15.67 ± 1.53 items/L in the wet season. Although there was an overall increase in mean abundance during the wet season in most rivers, the difference was not statistically significant.

Two-way ANOVA showed that river had a significant effect on the abundance of microplastic (*F*₃,₁₆ = 13.18, *p* < 0.001), but both season (*F*₁,₁₆ = 2.62, *p* = 0.125), and river and season interaction (*F*₃,₁₆ = 0.38, *p* = 0.765) were not significant. According to Tukey’s post hoc test, the abundance of microplastics in the Miri River was significantly greater than that of the Baram, Sibuti, and Niah rivers (*p* < 0.05), and the three rural rivers did not differ significantly. These results highlight the influence of land use and source of pollution on the spatial and seasonal variation in microplastic abundance in the rivers.

Besides patterns at the river scale, site-level variability was also observed, where the abundance of microplastics across sampling points (SP1–SP12) ranged from 6.33 ± 1.53 to 27.67 ± 2.31 items/L (Table S2). For example, in some sampling points such as SP4 (Miri River) and SP10 (Sibuti River), the abundance of microplastics was lower in the wet season than in the dry season, whereas in other locations (e.g., SP6 and SP12), the abundance of microplastics was relatively higher. This localized decrease during the wet season could be attributed to dilution and downstream transport caused by increased river discharge.

Additionally, across seasons, downstream samples observed in SP3 (Baram River) (10.67 ± 2.31–12.67 ± 1.53 items/L), SP6 (Miri River) (25.67 ± 2.52–27.67 ± 2.31 items/L), and SP12 (Niah River) (13.00 ± 1.00–17.67 ± 1.53 items/L) were higher than their upstream samples, respectively. These differences show that there is no uniform distribution of microplastics within the rivers.

### Physical and chemical characteristics of microplastics

**Fig. 3:**
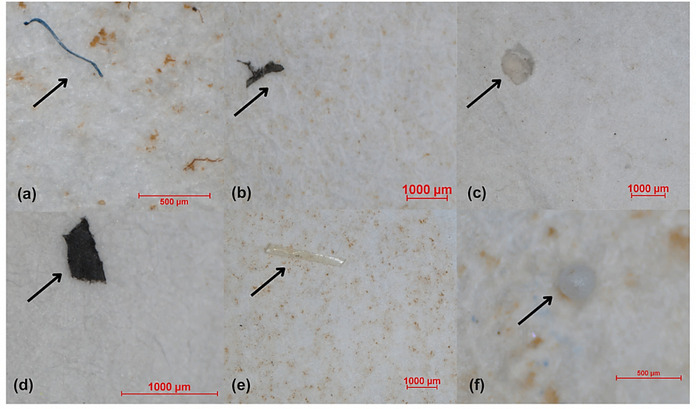
Selected microscopic images of identified microplastics in the surface water Baram, Miri, Sibuti, and Niah rivers, Malaysia: **a** fiber, **b** film, **c** foam, **d**, **e** fragment, and **f** bead

**Fig. 4:**
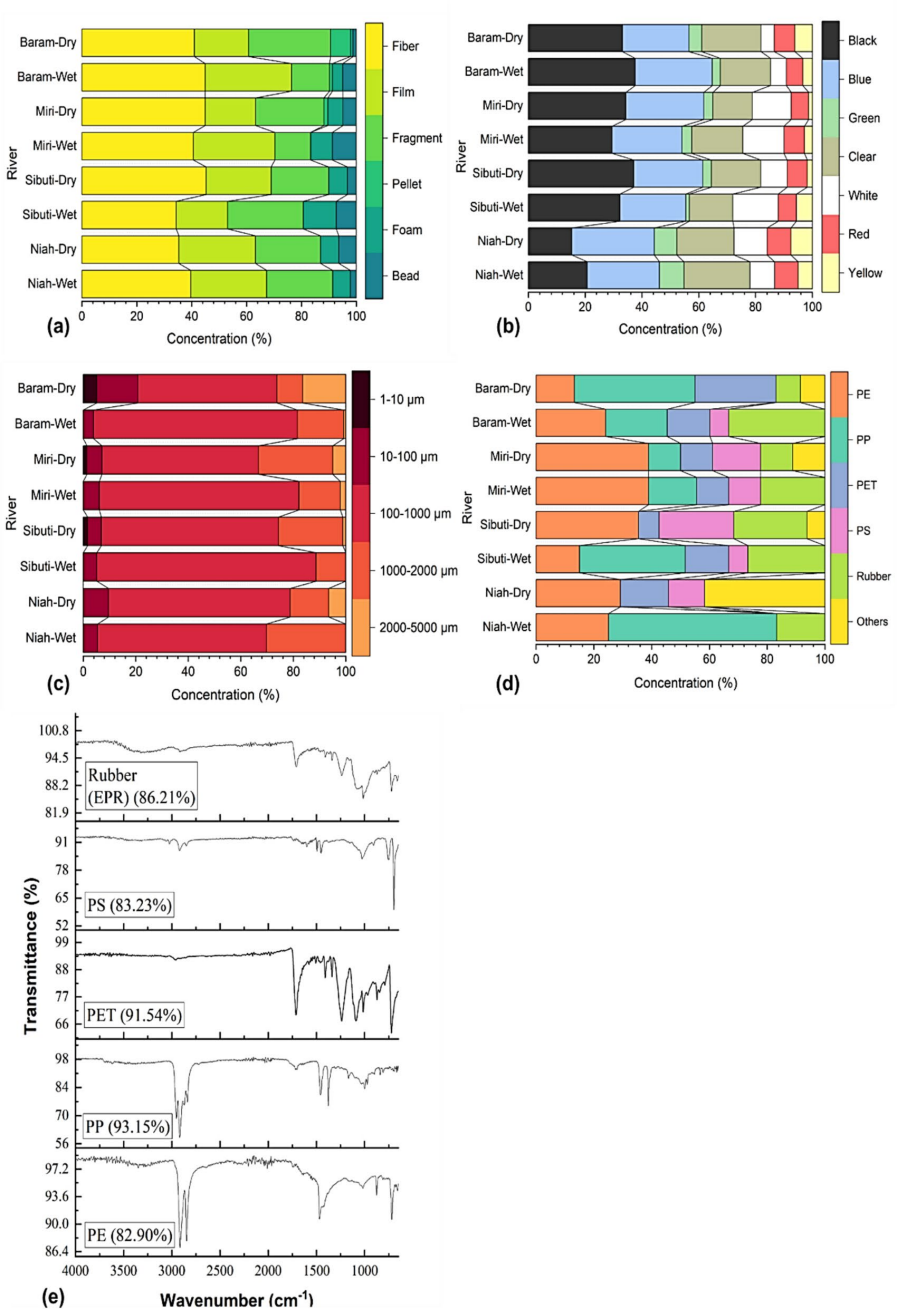
Dominant characteristics of microplastics according to **a** shape, **b** color, **c** size, **d** polymer, and **e** infrared spectra in the surface water of Baram, Miri, Sibuti, and Niah rivers during dry and wet seasons

Microscopic analysis confirmed the presence of various morphologies of microplastics in the surface waters of all rivers. Fibers, films, foam, fragments, and beads that were observed in the samples are represented in Fig. 3a–f. Fibers represented the most common shape group across all rivers and seasons, accounting for 34–45% of total particles (Table S2). Meanwhile, films and fragments accounted for 18–31% and 13–30% of total particles, respectively. Pellets (0–7%), foam (1–12%), and beads (1–9%) were in relatively lower proportions according to location and season (Fig. 4a).

The distribution of color differed among rivers and seasons, and the dominant colors were black (15–38%), blue (23–29%), and clear (14–23%) (Fig. 4b). Meanwhile, green, white, red, and yellow colors appeared in lower percentages at 1–9%, 5–16%, 6–8%, and 1–8%, respectively. The classification based on size showed that most of the microplastics had a size range of 100–1000 µm (53–84%) (Fig. 4c), and the least abundant size range was 1–10 µm at 0–5%. During the wet season, a decreasing trend of larger microplastics ranging from 2000–5000 µm in sizes was observed.

A representative sample (*n* = 134) of the particles was analyzed with ATR-FTIR, which confirmed the presence of polyethylene (PE) and polypropylene (PP) as the dominant microplastic polymers in the study areas at 13–39% and 0–58% of the total, respectively. Other microplastic polymers found were polyethylene terephthalate (PET), polystyrene (PS), and rubber-type particles at 0–28%, 0–26%, and 8–33%, respectively (Fig. 4d). The composition of polymers in the studied rivers was not uniform throughout the seasons. For example, PET and PS particles were absent in the Niah River during the wet season. The infrared spectra of dominating microplastic polymers in the surface water in the rivers are displayed in Fig. 4e.

The spectra of dominant microplastics were identified as PE due to the C–H stretch at 2915 cm^−1^ and 2844 cm^−1^, CH_2_ bend at 1466 cm^−1^ and 1461 cm^−1^, CH_3_ bend at 1375 cm^−1^, and CH_2_ rock at 730 cm^−1^ and 717 cm^−1^ (Jung et al., 2018). Meanwhile, microplastics that showed spectral results with peaks at 2917 cm^−1^ and 2837 cm^−1^ (C–H stretch), 1454 cm^−1^ (CH_2_ bend), 1375 cm^−1^ (CH_3_ bend), 1167 cm^−1^ (CH bend, CH_3_ rock, C–C stretch), 995 cm^−1^ (CH_3_ rock, CH_3_ bend, CH bend), 973 cm^−1^ (CH_3_ rock, C–C stretch), 839 cm^−1^ (CH_2_ rock, C–CH_3_ stretch), and 809 cm^−1^ (CH_2_ rock, C–C stretch, C–CH stretch) were identified as PP (Jung et al., 2018).

### Correlation of microplastic abundance with river water physicochemical and hydrological parameters

**Fig. 5:**
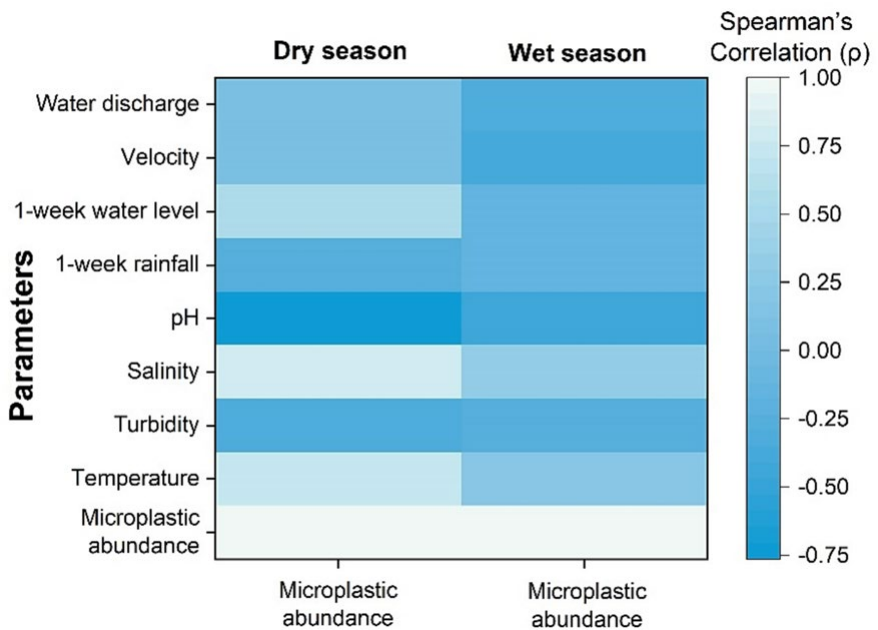
Spearman’s rank correlation coefficients (*ρ*) of microplastic abundance with river water physicochemical and hydrological parameters

The physicochemical and hydrological conditions of the studied rivers were observed to vary seasonally (Table S3-S5). The result of the Spearman’s rank correlation analysis revealed that the abundance of microplastics was significantly correlated with selected river water physicochemical parameters in the dry season (Table 1). Microplastic abundance showed a strong positive correlation with salinity (*ρ* = 0.799, *p* = 0.002) and temperature (*ρ* = 0.708, *p* = 0.010) and a strong negative correlation with pH (*ρ* = −0.764, *p* = 0.004) (Fig. 5).

**Table 1:** Significant levels (*p*) from Spearman’s rank correlation analysis between microplastic abundance in surface water and physicochemical and hydrological parameters during the dry and wet seasons

Parameter	(Dry season) *p*-value*	(Wet season) *p*-value*
Temperature	0.010	0.571
Turbidity	0.264	0.359
Salinity	0.002	0.296
pH	0.004	0.170
1-week hourly rainfall	0.436	0.589
1-week hourly water level relative to flood gauge	0.062	0.589
Velocity	0.762	0.230
Water discharge	0.795	0.318

*Significant correlations (*p* < 0.05)

In contrast, there were no significant correlations between the abundance of microplastics and turbidity, 1-week hourly rainfall, 1-week water level relative to the flood gauge, velocity, or water discharge during the dry season (*p* > 0.05). Additionally, the river water physicochemical and hydrological parameters that were examined in the wet season did not have significant correlations with microplastic abundance (*p* > 0.05). Overall, the outcomes of the correlation indicate that the relationships between microplastics and environmental parameters were weak and season-specific.

### Preliminary risk assessments of microplastic contamination

**Fig. 6:**
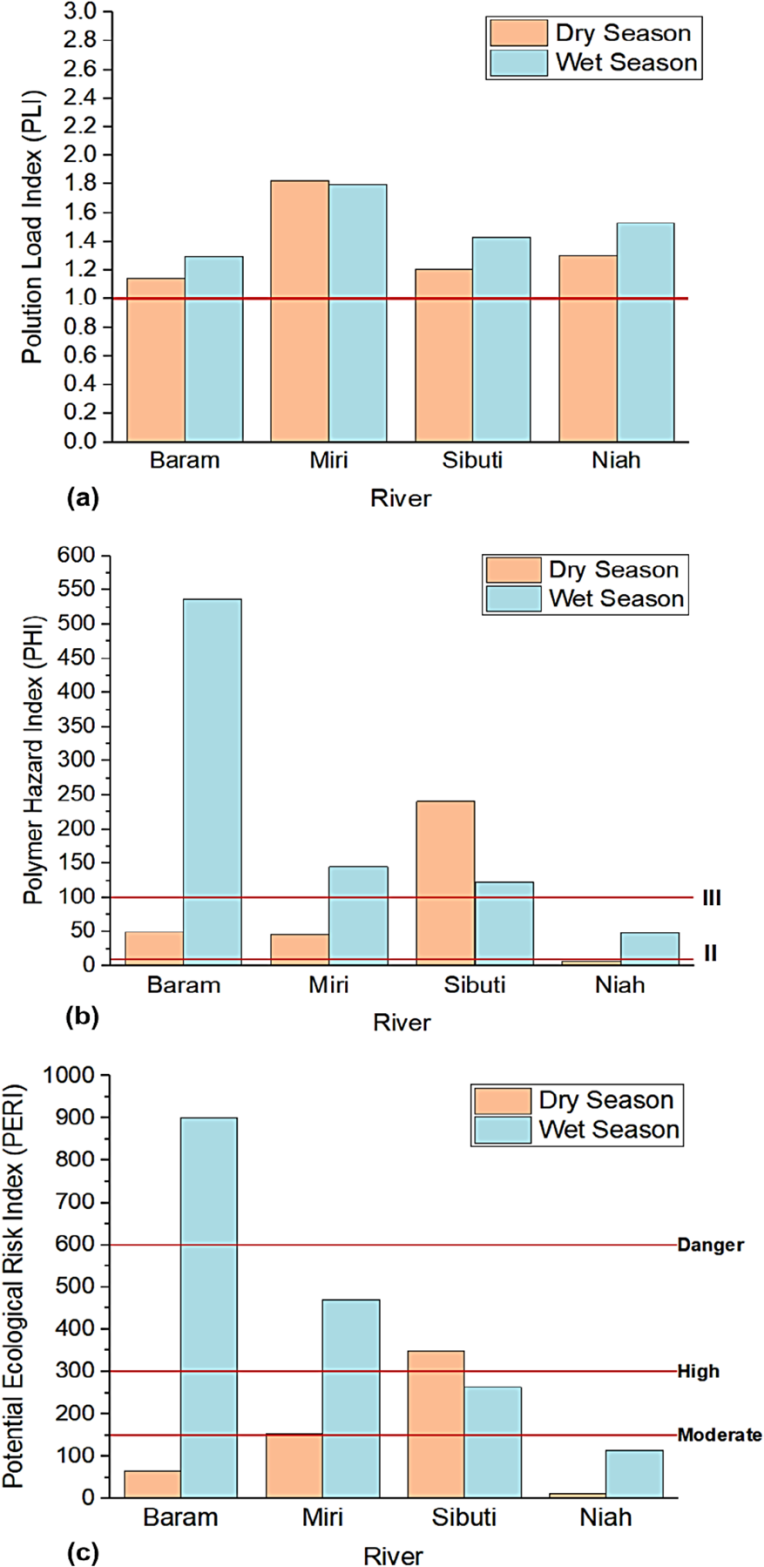
Preliminary risk assessments depicting **a** PLI, **b** PHI, and **c** PERI values involving microplastics in the surface water of Baram, Miri, Sibuti, and Niah rivers during dry and wet seasons

Comparison of ecological risk assessment of microplastic contamination of the Baram, Miri, Sibuti, and Niah rivers during dry and wet periods reveals a significant difference in PLI, PHI, and PERI, as shown in Fig. 6. Site-specific PLI values confirmed the significant level of microplastic pollution along the rivers (1.47–2.07) (Table S6). The PLI_River_ for the Baram River was 1.15 during the dry season, and it increased to 1.30 in the wet season. Miri River had the highest PLI of 1.83 in the dry season, decreasing slightly to 1.80 in the wet season (Fig. 6a). The PHI values fluctuated due to the presence of rubber with a high hazard score of 1628 (Lithner et al., 2011). For example, the PHI_River_ of Baram River reached 536.46 during the wet season, and the Sibuti River had a PHI_River_ of 240.19 in the dry season (Fig. 6b), both labelled Category IV (danger level) (Baycan et al., 2025; Lithner et al., 2011; Ziembowicz & Kida, 2025).

Overall, the PERI_River_ values during the wet season were generally higher compared to the dry season. PHI_River_ trends mirrored in PERI_River_ value, where the Baram River reached 899.80 (danger-level) during the wet season, compared to 469.73 (danger-level) in the Miri River (Fig. 6c). The highest and lowest PHI were observed at 707.43 (Category IV) in SP10 (Sibuti River) during the wet season and 2.71 (Category I) in SP2 (Baram River) during the dry season, respectively. Similarly, the lowest PERI value of 3.81 (low risk) was recorded during the dry season in SP2 (Baram River), and the highest PERI was 2274.35 (extreme danger) during the wet season in SP6 (Miri River) (Table S6). SP6, which is located at the river mouth in Miri River, is significantly affected by human activities and pollutants from different sources along the river (fishing, commercial, and residential activities).

Due to the ATR-FTIR limitations, there was a lack of wet season polymer composition data in SP8 (Sibuti River), resulting in high variation in PHI and PERI values. For example, despite being in the river mouth and a relatively high PLI value of 1.63, SP12 in the Niah River exhibited Category II PHI (4.33–5.00), and low-risk PERI (9.77–11.51) compared to the Category IV PHI (10.25–545.00) and danger-level PERI (14.90–1120.05) in the upstream SP11, underscoring the need for polymer-aware risk assessments.

## Discussion

### Land-use influences and comparison with previous studies

Microplastic abundance varied among rivers but not seasons according to two-way ANOVA, which indicated that spatial differences influenced by land use and human activities are more significant than seasonal variability. However, limited sample size may have reduced the statistical power to detect subtle seasonal effects. The higher abundance in urbanized Miri River compared to rural Baram, Sibuti, and Niah rivers is consistent with previous studies (Kunz et al., 2023; Wagner et al., 2019).

Compared with other Malaysian freshwater studies, the microplastic abundance in the Miri River was relatively lower than that of the Perak River (Hassan et al., 2025) (Table 2), owing to the larger catchment area (15,151 km^2^), which covers 70% of the state, and the stronger anthropogenic pressure of the Perak River. The microplastics in the Miri River likely originated from the residential and commercial areas (SP4, SP6), and port-related activities (SP5), similar to the findings in the Pekalongan River estuary in Indonesia (Ismanto et al., 2023), Singapore Straits (Curren & Leong, 2023), and the Yangshui River in China (Biao et al., 2025).

**Table 2:** Comparison between microplastics abundance in the surface water of the current study and previous studies (based on the bucket-based collection method)

Location	Microplastic abundance (items/L)	Dominant microplastic shape	Dominant microplastic size	Dominant microplastic color	Dominant microplastic polymer**	Reference
Baram River, Malaysia	8.89 ± 1.35–11.22 ± 1.91	Fiber	100–1000 µm	Black, blue	PP	This study
Miri River, Malaysia	22.33 ± 3.39–22.44 ± 3.01	Fiber	100–1000 µm	Black, blue	PE	This study
Sibuti River, Malaysia	10.17 ± 2.32–13.75 ± 2.95	Fiber	100–1000 µm	Black, blue	PE	This study
Niah River, Malaysia	11.33 ± 1.26–15.67 ± 1.53	Fiber	100–1000 µm	Blue, clear	PP	This study
Perak River, Malaysia	200 ± 27.22–311 ± 8.19	Granules, irregular particles	1–10 µm	Not mentioned	Rayon, EPDM	Hassan et al. (2025)
Dungun River, Malaysia	0.023–0.30	Fiber	< 200 µm	Black, transparent	PP, PAN, rayon	Tee et al. (2020)
Singapore Straits	143–196	Fragment	< 500 µm	Clear	Not mentioned	Curren & Leong (2023)
Cisadane River, Indonesia	0.0133–0.113*	Fragment	500–1000 µm	Not mentioned	PE, PS, PP	Sulistyowati et al. (2022)
Pekalongan River, Indonesia	45.2 ± 1.3–99.1 ± 1.6	Fragment	300–1000 µm	Black, blue	PS, PET, PA	Ismanto et al. (2023)
U-Taphao, Thailand	0.24 ± 0.11–0.41 ± 0.08	Fiber	> 500–1000 µm	Blue	PE, PP, PET	Pradit et al. (2023)
Eight protected area in Western Complex, Thailand	0.30 ± 0.32	Fiber	50–500 µm	Blue, black	PP, PET	Teampanpong et al. (2024)
Saigon River, Vietnam	0.000160–0.00159*	Fiber	1000–2800 µm	White, transparent, blue, green	PE, PP, EVA	Nguyen et al. (2022)
Bach Hac-Red River confluence, Vietnam	0.0432–0.103*	Fiber	< 100 µm	Not mentioned	PET	Le et al. (2026)
Cagayan de Oro River, Philippines	0.30*	Fiber	300–500 µm	Blue	Polyacetylene	Gabriel et al. (2023)
Five river mouths of Manila Bay, Philippines	1.58–57.7*	Fragment	250–5000 µm	White, transparent, blue	PP, LDPE, HDPE, PS	Osorio et al. (2021)
Yanshui River, China	199.2 ± 141.5–249.6 ± 100.6	Particle	< 100 µm	Not mentioned	PP	Biao et al. (2025)
Sabaki River, Kenya	0.120–0.264*	Fiber	≤ 2000 µm	White	PE, PET, PP	Kamau et al. (2024)
Godavari River, India	3.5–15.0	Fiber	< 500 µm	Blue, transparent	PC, PVC, PP, nylon	Sekar and Sundaram (2025)

*Values converted from items/m^3^ to items/L for comparison with the current study

**PP Polypropylene, *PE* Polyethylene, *EPDM* Ethylene propylene diene monomer, *PAN* Polyacrylonitrile, *PS* Polystyrene, *EVA* Ethylene-vinyl acetate, *PET* Polyethylene terephthalate, *PA* Polyamide, *LDPE* Low-density polyethylene, *HDPE* High-density polyethylene, *PC* Polycarbonate, *PVC* Polyvinyl chloride

In contrast, the Baram River has lower values compared to the Sibuti and Niah rivers despite having a larger river catchment and the presence of small-scale residential areas (SP3), wood-based industries (SP1), and shipping and docking infrastructure (SP2). The patterns observed suggest a larger catchment area may promote dilution and downstream particle dispersion. A similar finding was observed in the Saigon River in Vietnam, where lower abundance was observed compared to the nearby smaller canals, which might be caused by the dilution of the higher flow of the river in a larger area (Nguyen et al., 2022).

The intermediate microplastic abundance observed in the Sibuti River in this study reflects the mixed land use and fishing activities, agriculture, and mangrove forest reserve between the river mouth and upstream sites, influencing microplastic contamination. Mangrove acts as a natural filter that traps pollutants (Valsan et al., 2024), suggesting the lower microplastic abundance in the river mouth (SP10) compared to the upstream sampling points (SP7–SP9) was due to the entrapment of particles by mangroves. The presence of microplastics in the Niah River that flows through a fishing village and agricultural lands can be attributed to agricultural runoff and localized sources of microplastic pollution, like fishing gear (Teampanpong et al., 2024; Tee et al., 2020).

The downstream samples in the Baram (SP3), Miri (SP6), and Niah (SP12) rivers exhibited higher abundance of microplastics compared to upstream samples, similar to the findings in the Godavari River in India (Sekar & Sundaram, 2025). Teampanpong et al. (2024) reported that areas with lower elevation within the Western Forest Complex of Thailand tend to promote the accumulation of microplastics (*p* < 0.05), due to denser human populations and low water flow.

### Microplastic characteristics and potential sources

Fibers and fragments were the common microplastic shapes in the current study and other freshwater studies (Kamau et al., 2024; Le et al., 2026; Sulistyowati et al., 2022). Fibers are usually released during the abrasion and washing of synthetic textiles and fishing equipment (Napper & Thompson, 2016; Teampanpong et al., 2024; Tee et al., 2020), reflecting the residential and fishing activities in the rivers. Formation of fragments is likely due to the weathering of larger plastic items (Sekar & Sundaram, 2025) due to mismanaged waste.

Other microplastic shapes, such as films, beads, and foams, are associated with urban and commercial sources, such as plastic bags, personal care products, and Styrofoam packaging (anak Alexander Tampang & Mohan Viswanathan, 2022; Le et al., 2026; Sekar & Sundaram, 2025). Pellets were only observed in the Baram and Miri rivers and may have originated from raw plastic materials spilled from container ships, abrasives in body care products and cleansing agents (Curren & Leong, 2023; Kamau et al., 2024; Liong et al., 2021).

The dominance of microplastics in the size class of 100–1000 µm aligns with the findings of other river systems, which reported high levels of microplastics < 1000 µm (Ismanto et al., 2023; Pradit et al., 2023; Sulistyowati et al., 2022). Microplastics may undergo fragmentation by wind, tides, wave action, and vertical water mixing and become smaller (Sekar & Sundaram, 2025). A contrasting pattern was observed in the Sabaki River in Kenya, where a larger microplastic size range dominated the wet season (Kamau et al., 2024). The reduced abundance of larger microplastics (2000–5000 µm) during the wet season is likely caused by the increased water flow and turbulence, which disperses larger particles downstream.

Black, blue, and clear microplastics were prevalent in this study, which were consistent with the findings in Dungun River in Malaysia, Pekalongan River in Indonesia, and Godavari River in India (Ismanto et al., 2023; Sekar & Sundaram, 2025; Tee et al., 2020). Microplastic color can predict their sources. Blue and transparent colors are commonly seen in plastic bags, food packaging, and fishing nets and lines (Gabriel et al., 2023; Sekar & Sundaram, 2025; Tee et al., 2020) while black color is associated with vehicle tires, plastic mulch, and garbage bags (Teampanpong et al., 2024). However, UV exposure can alter color over time (Sekar & Sundaram, 2025), while black particles may persist in the environment due to their higher UV resistance (Kamau et al., 2024).

The predominance of PP and PE microplastic polymers in the current study aligns with findings from other freshwater systems (Biao et al., 2025; Kamau et al., 2024; Nguyen et al., 2022; Pradit et al., 2023; Sulistyowati et al., 2022; Tee et al., 2020). PP and PE are also the most produced consumer plastics globally (PlasticsEurope, 2025). PE likely originated from single-use packaging and plastic bags (Sekar & Sundaram, 2025; Valsan et al., 2024), used daily by the locals near the commercial and waterfront areas (SP4, SP6) in the Miri River. PS and foam particles are linked to disposable food containers and storage boxes (Choong et al., 2021; Liong et al., 2021), which are commonly used for food takeaway and fish vendors for storing and transporting fresh catch within the area.

Conversely, the Baram River showed higher PP polymers, which are typically connected with ropes, fishing equipment, and packaging (Le et al., 2026; Sekar & Sundaram, 2025). The PP polymer was likely derived from ropes used in shipping and docking activities in SP2. The lower densities of PE (0.91–0.96 g/cm^3^) and PP (0.89–0.94 g/cm^3^) (GESAMP, 2019; Nguyen et al., 2022) may enable them to float and travel long distances in the water column.

In the Sibuti and Niah rivers, the observed PE, PP, PET, and rubber particles are linked with agriculture, mangroves, and fishing communities. PP polymer is used in plastic mulch (Baycan et al., 2025) while PET links to household wastewater and synthetic textiles (Choong et al., 2021), linking to the land use in SP8–SP11. Meanwhile, rubber particles are generated from tire wear (Ziembowicz & Kida, 2025) and can enter the river through runoff during rain (Kamau et al., 2024).

The absence of PET and PS in the Niah River and during the wet season suggests dilution due to rainwater input. However, the density of microplastics is not constantly uniform due to the influence of adsorption, microbial colonization, and biofouling, which can alter their environmental behavior (Sekar & Sundaram, 2025). These results suggest that the distribution of microplastics in river systems is influenced by the seasonal hydrodynamics of the river, and not solely by the source inputs.

### Influence of physicochemical and hydrological conditions on microplastic distribution

The microplastic abundance had insignificant relationships with most of the studied river water physicochemical parameters, similar to other studies in the Langat River in Malaysia (Ali et al., 2026). In this study, salinity was strongly positively correlated (*p* = 0.002) with the abundance of microplastics in the dry season, reflecting saltwater intrusion during the dry season and longer particle retention time due to reduced freshwater discharge, a pattern similarly reported in another tropical river-estuary environment (Kamau et al., 2024). These processes increase stratification and decrease vertical mixing, which promotes the retention of particles in surface waters.

The temperature also showed a positive correlation (*p* = 0.010) with the abundance of microplastics in the dry season. This observation is similar to the study in the Karnaphuli River, where temperature influences the hydrodynamics mechanisms of microplastics as well as their fragmentation (Rakib et al., 2023). In contrast, pH had a strong negative correlation (*p* = 0.004) with microplastic abundance during the dry season, indicating that if the pH value in the studied rivers is low, there is a high abundance of microplastics. Microplastics retain the electrostatic force of attraction at low pH, which causes the aggregation of microplastics and facilitates deposition from the water column (Kumar et al., 2021).

In contrast, there were no significant correlations among turbidity, rainfall, river depth, velocity, and water discharge, indicating that short-term hydrological measurements are not able to capture microplastic transport behavior. This finding contrasts with the study by Curren and Leong (2023), where greater rainfall resulted in greater surface runoff from the land-based sources, resulting in significantly higher microplastics input in water bodies. Additionally, no meaningful correlations were found during the wet season as the hydrological variability was too high. Rainfall and runoff increase dilution, mixing, and episodic inputs of microplastics and may mask stable spatial relationships. Overall, the processes of microplastic transport are controlled by interactions between hydrodynamic and estuarine processes instead of simple environmental relationships.

### Potential microplastic implications in river systems

In this study, the PLI values across sampling points and seasons were above 1.00, indicating the significant presence of microplastic contamination (Chen et al., 2026; Le et al., 2026; Tomlinson et al., 1980). The preliminary ecological risk assessments revealed spatial variability in the microplastic contamination in the studied rivers. In the current study, the Baram River had higher PERI_River_ during the wet season despite the Miri River having the highest overall microplastic abundance and stronger urban influence.

Although rubber accounted for small proportions of the total polymer composition, its high hazard score (hazard score = 1628) drove up the PHI values in certain sampling points. Kong et al. (2024) reported a similar observation, where the presence of PVC (hazard score = 10,551) polymers significantly increased the PERI values in the rural Longjiang River in China. Thus, pollution levels should not be the sole indicator of the current status but provide a reference for the possible ecological hazards of microplastic toxicity. Within the present study, site-level variability indicates the presence of localized hotspots.

Compared with other freshwater systems, the PLI_River_ and PERI_River_ values of the urbanized Miri River were lower than those reported in the Yangshui River, China (PLI: 1.8–4.8, PERI: 296–5400), due to higher industrial and urban pressures (Biao et al., 2025), but were comparable to the Bach Hac-Red River confluence, Vietnam, which shows low to medium risk of PHI (1.88–127.37) and PERI (4.31–459.83) indices (Le et al., 2026). Meanwhile, the PLI_River_, PHI_River_, and PERI_River_ values of the rural river Baram, Sibuti, and Niah were lower than those reported in the Longjiang River (PLI: 1.01–10.51, PHI: 11.0–10,551) (Kong et al., 2024). The Longjiang River has a larger river catchment (16,878 km^2^) than Sibuti and Niah rivers and flows through highly cultivated agricultural lands exceeding 7530 km^2^, where intensive use of agricultural PVC films increased microplastic input and polymer-based risk index.

Microplastics pose a threat to aquatic life, including fish, invertebrates, and benthic fauna, through ingestion and bioaccumulation. Certain colors such as blue attract fish, leading to ingestion (Ory et al., 2017). Microplastic effects in aquatic animals span from impaired feeding, growth, survival, and reproduction, altered behavior and swimming patterns, gut blockages, inflammation, oxidative stress, and endocrine disruption (Pal et al., 2024).

High levels of microplastics in fishery areas and river mouths indicate the possibility of dietary exposure in humans, especially by eating contaminated seafood, which can cause functional disorders (Curren et al., 2020; Li et al., 2023). Human health risks may be further increased as microplastic polymers such as PE and PP contain hazardous additives and can adsorb pollutants (Li et al., 2023; Pal et al., 2024). Overall, quantitative frameworks of risk assessment of ecological risks can help inform monitoring and mitigation strategies in tropical river systems using risk indices like PLI, PHI, and PERI.

## Conclusion

This study evaluates the microplastic pollution in four Sarawak rivers in Malaysia, focusing on the spatial-temporal distributions, particle characteristics, environmental relationships, and ecological risks. All rivers contained microplastics, and spatial variation between them was significant due to anthropogenic pressure. In contrast, there was no significant difference in seasonal microplastic abundance.

Physicochemical and hydrological parameters showed limited relations with microplastic abundance. The PLI, PHI, and PERI ecological risk indices confirmed widespread contamination across all rivers and seasons, with higher risk levels generally associated with urbanized and downstream locations. Overall, the distribution and ecological risk of microplastics in the studied rivers are driven by spatial human pressures, and not by seasonal variation, which forms the basis of information on future monitoring and management.

## Supplementary Information

Below is the link to the electronic supplementary material.

ESM 1 (47.6 KB DOCX)

## Data Availability

All data used during this study are included in the paper, the supplemental information file, and/or are available from the corresponding author on reasonable request.
