# Supplementary material for: Spatiotemporal distribution of microplastics and associations with river water physicochemical and hydrological parameters, including risk assessments in four tropical rivers in Sarawak, Malaysia
Source: Environ Monit Assess. 2026 Jun 13;198(7):713. doi: 10.1007/s10661-026-15554-0 (PMC13264592; doi:10.1007/s10661-026-15554-0)
Supplement: Supplementary file 1 — (47.6 KB DOCX) [file 10661_2026_15554_MOESM1_ESM.docx]

# Supplementary Material

**Table S1** Coordinates and site descriptions of sampling locations in the Baram, Miri, Sibuti, and Niah rivers, in Sarawak, Malaysia

| River | Sampling point | Site description | Latitude | Longitude |
| --- | --- | --- | --- | --- |
| Baram | SP1 | Near plywood industry and sawmill | 4°34'19.5"N | 114°01'30.6"E |
|  | SP2 | Near shipping service and dockyard | 4°34'42.7"N | 113°59'14.4"E |
|  | SP3 | Near river mouth, small residential area | 4°34'49.1"N | 113°58'57.4"E |
| Miri | SP4 | Near fishmarket and residential area | 4°26'32.0"N | 114°00'12.0"E |
|  | SP5 | Near ship port | 4°24'14.3"N | 113°59'24.3"E |
|  | SP6 | Near river mouth, fishermen’ jetties, residential area and commercial area | 4°24'00.2"N | 113°59'11.1"E |
| Sibuti | SP7 | Near jetty, opposite Subis center | 4°03'23.2"N | 113°50'50.2"E |
|  | SP8 | Near jetty and agricultural lands | 4°02'57.4"N | 113°49'29.9"E |
|  | SP9 | Near village | 4°02'32.1"N | 113°48'41.0"E |
|  | SP10 | Near river mouth and fishermen village | 3°59'30.4"N | 113°43'38.1"E |
| Niah | SP11 | Near fishermen village | 3°56'18.7"N | 113°41'37.2"E |
|  | SP12 | Near river mouth | 3°57'33.3"N | 113°41'59.7"E |

**Table S2** Spatiotemporal abundance and shape proportions of microplastics in the surface water of Baram, Miri, Sibuti and Niah rivers in Malaysia

| Season | River | Sampling point | Microplastic abundance (items/L) | Fiber (%) | Film (%) | Fragment (%) | Pellet (%) | Foam (%) | Bead (%) |
| --- | --- | --- | --- | --- | --- | --- | --- | --- | --- |
| Dry | Baram | SP1 | 6.67 ±1.15 | 40.28 | 15.28 | 29.17 | 15.28 | 0.00 | 0.00 |
| Wet | Baram | SP1 | 9.67 ± 1.15 | 47.81 | 34.34 | 11.11 | 3.03 | 3.70 | 0.00 |
| Dry | Baram | SP2 | 9.33 ± 0.58 | 39.63 | 28.52 | 24.44 | 3.70 | 0.00 | 3.70 |
| Wet | Baram | SP2 | 11.33 ± 3.06 | 42.06 | 31.35 | 14.48 | 0.00 | 2.78 | 9.33 |
| Dry | Baram | SP3 | 10.67 ± 2.31 | 43.06 | 15.28 | 36.11 | 2.78 | 2.78 | 0.00 |
| Wet | Baram | SP3 | 12.67 ± 1.53 | 44.82 | 28.69 | 15.95 | 0.00 | 4.95 | 5.59 |
| Dry | Miri | SP4 | 17.67 ± 3.51 | 42.33 | 24.87 | 23.02 | 1.59 | 3.17 | 5.03 |
| Wet | Miri | SP4 | 14.33 ± 3.51 | 44.13 | 29.08 | 14.00 | 0.00 | 4.88 | 7.91 |
| Dry | Miri | SP5 | 24.00 ± 3.00 | 44.78 | 22.49 | 24.12 | 0.00 | 4.41 | 4.21 |
| Wet | Miri | SP5 | 25.00 ± 4.36 | 40.44 | 30.68 | 13.03 | 0.00 | 8.14 | 7.71 |
| Dry | Miri | SP6 | 25.67 ± 2.52 | 47.57 | 7.84 | 27.36 | 2.73 | 9.03 | 5.46 |
| Wet | Miri | SP6 | 27.67 ± 2.31 | 37.29 | 29.24 | 12.05 | 0.00 | 10.71 | 10.71 |
| Dry | Sibuti | SP7 | 8.67 ± 2.08 | 50.27 | 19.16 | 24.51 | 0.00 | 3.03 | 3.03 |
| Wet | Sibuti | SP7 | 17.33 ± 2.08 | 39.04 | 15.26 | 28.67 | 0.00 | 11.66 | 5.36 |
| Dry | Sibuti | SP8 | 6.33 ± 1.53 | 52.22 | 13.89 | 16.39 | 0.00 | 10.83 | 6.67 |
| Wet | Sibuti | SP8 | 11.67 ± 3.79 | 31.99 | 19.95 | 26.11 | 0.00 | 12.82 | 9.12 |
| Dry | Sibuti | SP9 | 10.67 ± 2.08 | 40.97 | 28.03 | 18.06 | 0.00 | 9.60 | 3.33 |
| Wet | Sibuti | SP9 | 13.67 ± 2.89 | 31.21 | 20.59 | 29.25 | 0.00 | 15.03 | 3.92 |
| Dry | Sibuti | SP10 | 15.00 ± 3.61 | 37.58 | 33.65 | 24.83 | 0.00 | 3.94 | 0.00 |
| Wet | Sibuti | SP10 | 12.33 ± 3.06 | 35.04 | 19.20 | 26.21 | 0.00 | 8.49 | 11.05 |
| Dry | Niah | SP11 | 9.67 ± 1.53 | 34.62 | 27.42 | 24.39 | 0.00 | 6.06 | 7.50 |
| Wet | Niah | SP11 | 13.67 ± 1.53 | 41.51 | 27.14 | 21.75 | 0.00 | 7.22 | 2.38 |
| Dry | Niah | SP12 | 13.00 ± 1.00 | 36.05 | 28.33 | 22.95 | 0.00 | 7.33 | 5.34 |
| Wet | Niah | SP12 | 17.67 ± 1.53 | 37.74 | 28.22 | 26.36 | 0.00 | 5.59 | 2.08 |

**Table S3** Summary of seasonal water physicochemical and hydrological readings in Baram, Miri, Sibuti and Niah rivers

| River | Season | Mean temperature (°C) | Mean turbidity (NTU) | Mean salinity (ppt) | Mean pH | Mean 1-week hourly rainfall (mm) | Mean 1-week hourly water level relative to flood gauge (m) | Mean velocity (m/s) | Water discharge (m³/s) |
| --- | --- | --- | --- | --- | --- | --- | --- | --- | --- |
| Baram | Dry | 31.68 ± 2.14 | 33.08 ± 15.93 | 3.42 ± 1.13 | 8.52 ± 0.63 | 0.00 ± 0.00 | 0.80 ± 0.27 | 0.12 ± 0.01 | 236.36 ± 120.67 |
| Baram | Wet | 28.70 ± 0.48 | 306.43 ± 232.39 | 0.28 ± 0.24 | 7.52 ± 0.46 | 0.22 ± 0.83 | 2.92 ± 0.34 | 0.18 ± 0.05 | 728.40 ± 339.43 |
| Miri | Dry | 36.52 ± 1.52 | 35.87 ± 13.77 | 9.93 ± 3.57 | 7.05 ± 0.30 | 0.00 ± 0.00 | 1.02 ± 0.40 | 0.13 ± 0.02 | 74.21 ± 5.90 |
| Miri | Wet | 30.23 ± 1.01 | 49.90 ± 14.18 | 5.22 ± 1.58 | 6.00 ± 0.36 | 0.21 ± 0.93 | 1.35 ± 0.52 | 0.15 ± 0.04 | 55.79 ± 14.99 |
| Sibuti | Dry | 32.64 ± 3.07 | 59.28 ± 32.15 | 1.10 ± 2.16 | 7.99 ± 0.73 | 0.41 ± 2.48 | 0.27 ± 0.43 | 0.12 ± 0.01 | 29.14 ± 6.69 |
| Sibuti | Wet | 31.38 ± 1.92 | 102.78 ± 10.29 | 0.29 ± 0.47 | 7.83 ± 0.35 | 0.47 ± 2.32 | - 0.22 ± 0.96* | 0.16 ± 0.06 | 26.62 ± 15.29 |
| Niah | Dry | 32.68 ± 0.46 | 112.25 ± 60.18 | 0.36 ± 0.44 | 7.30 ± 0.71 | 0.51 ± 2.87 | 0.58 ± 0.53 | 0.13 ± 0.01 | 43.46 ± 14.48 |
| Niah | Wet | 31.78 ± 0.18 | 212.23 ± 31.36 | 0.24 ± 0.28 | 7.75 ± 0.28 | 1.81 ± 10.38 | 1.10 ± 0.61 | 0.17 ± 0.04 | 29.72 ± 10.12 |

*Water level likely unreliable due to gauge error

**Table S4** Site-specific river water physicochemical and hydrological parameters during dry season at Baram, Miri, Sibuti, and Niah rivers, in Sarawak, Malaysia

| River name | Sampling point | Latitude | Longitude | Approximate river section width (m) | Mean river section depth (m) | Mean river section velocity (m/s) | Calculated water discharge (m³/s) | Water temperature (°c) | Water turbidity (NTU) | Water salinity (ppt) | Water pH |
| --- | --- | --- | --- | --- | --- | --- | --- | --- | --- | --- | --- |
| Baram River | SP1 | 4°34'19.5"N | 114°01'30.6"E | 441.69 | 5.30 | 0.12 | 363.79 | 29.20 | 23.20 | 2.62 | 9.30 |
|  | SP2 | 4°34'42.7"N | 113°59'14.4"E | 533.40 | 4.10 | 0.13 | 222.40 | 33.20 | 51.80 | 3.68 | 8.30 |
|  | SP3 | 4°34'49.1"N | 113°58'57.4"E | 611.66 | 2.47 | 0.11 | 122.88 | 32.30 | 24.30 | 3.93 | 8.00 |
| Miri River | SP4 | 4°26'32.0"N | 114°00'12.0"E | 87.69 | 4.07 | 0.15 | 79.86 | 34.60 | 31.80 | 5.62 | 7.30 |
|  | SP5 | 4°24'14.3"N | 113°59'24.3"E | 77.15 | 3.20 | 0.13 | 68.21 | 35.40 | 25.50 | 11.40 | 6.90 |
|  | SP6 | 4°24'00.2"N | 113°59'11.1"E | 111.71 | 2.67 | 0.11 | 74.57 | 37.40 | 51.20 | 12.70 | 6.80 |
| Sibuti River | SP7 | 4°03'23.2"N | 113°50'50.2"E | 43.50 | 2.55 | 0.14 | 35.64 | 29.40 | 92.10 | 0.00 | 8.90 |
|  | SP8 | 4°02'57.4"N | 113°49'29.9"E | 62.70 | 2.55 | 0.11 | 21.49 | 34.30 | 71.80 | 0.00 | 7.40 |
|  | SP9 | 4°02'32.1"N | 113°48'41.0"E | 69.60 | 2.60 | 0.12 | 33.80 | 31.20 | 59.40 | 0.00 | 8.40 |
|  | SP10 | 3°59'30.4"N | 113°43'38.1"E | 93.10 | 2.77 | 0.11 | 25.63 | 36.10 | 13.90 | 4.22 | 7.30 |
| Niah River | SP11 | 3°56'18.7"N | 113°41'37.2"E | 89.90 | 3.15 | 0.13 | 33.21 | 33.90 | 154.00 | 0.00 | 7.70 |
|  | SP12 | 3°57'33.3"N | 113°41'59.7"E | 128.10 | 2.38 | 0.12 | 53.70 | 32.70 | 70.60 | 1.10 | 6.80 |

**Table S5** Site-specific river water physicochemical and hydrological parameters during wet season at Baram, Miri, Sibuti, and Niah rivers, in Sarawak, Malaysia

| River name | Sampling point | Latitude | Longitude | Approximate river section width (m) | Mean river section depth (m) | Mean river section velocity (m/s) | Calculated water discharge (m³/s) | Water temperature (°c) | Water turbidity (NTU) | Water salinity (ppt) | Water pH |
| --- | --- | --- | --- | --- | --- | --- | --- | --- | --- | --- | --- |
| Baram River | SP1 | 4°34'19.5"N | 114°01'30.6"E | 473.57 | 8.40 | 0.23 | 904.99 | 29.40 | 45.60 | 0.01 | 8.10 |
|  | SP2 | 4°34'42.7"N | 113°59'14.4"E | 548.80 | 8.90 | 0.19 | 941.45 | 28.00 | 480.00 | 0.70 | 7.30 |
|  | SP3 | 4°34'49.1"N | 113°58'57.4"E | 636.56 | 4.13 | 0.13 | 338.76 | 28.20 | 395.00 | 0.25 | 7.20 |
| Miri River | SP4 | 4°26'32.0"N | 114°00'12.0"E | 82.93 | 4.70 | 0.19 | 72.69 | 29.80 | 45.80 | 4.00 | 6.40 |
|  | SP5 | 4°24'14.3"N | 113°59'24.3"E | 101.25 | 3.47 | 0.14 | 50.69 | 29.80 | 39.60 | 4.98 | 5.70 |
|  | SP6 | 4°24'00.2"N | 113°59'11.1"E | 122.89 | 2.87 | 0.12 | 43.98 | 31.70 | 65.70 | 6.92 | 6.00 |
| Sibuti River | SP7 | 4°03'23.2"N | 113°50'50.2"E | 58.10 | 2.20 | 0.13 | 16.68 | 29.40 | 101.00 | 0.05 | 8.20 |
|  | SP8 | 4°02'57.4"N | 113°49'29.9"E | 68.10 | 1.60 | 0.10 | 10.84 | 31.80 | 90.60 | 0.05 | 8.00 |
|  | SP9 | 4°02'32.1"N | 113°48'41.0"E | 90.10 | 2.10 | 0.24 | 45.46 | 33.30 | 104.00 | 0.05 | 8.00 |
|  | SP10 | 3°59'30.4"N | 113°43'38.1"E | 96.20 | 2.10 | 0.17 | 33.48 | 30.80 | 116.00 | 1.17 | 7.30 |
| Niah River | SP11 | 3°56'18.7"N | 113°41'37.2"E | 91.20 | 2.10 | 0.19 | 36.87 | 32.40 | 235.00 | 0.05 | 7.90 |
|  | SP12 | 3°57'33.3"N | 113°41'59.7"E | 137.00 | 1.20 | 0.14 | 22.56 | 31.20 | 190.00 | 0.50 | 7.50 |

**Table S6** Site-specific microplastic risk assessment indices (PLI, PHI, and PERI) for all sampling locations and seasons in Baram, Miri, Sibuti and Niah rivers, in Sarawak, Malaysia

| Season | River | Sampling point | Pollution load index (PLI) | Polymer hazard index (PHI) | Potential ecological risk index (PERI) |
| --- | --- | --- | --- | --- | --- |
| Dry | Baram | SP1 | 1.00 | 235.29 | 235.88 |
| Dry | Baram | SP2 | 1.18 | 2.71 | 3.81 |
| Dry | Baram | SP3 | 1.27 | 184.44 | 295.85 |
| Dry | Miri | SP4 | 1.63 | 545.17 | 1448.31 |
| Dry | Miri | SP5 | 1.90 | 11.33 | 40.90 |
| Dry | Miri | SP6 | 1.96 | 15.67 | 60.47 |
| Dry | Sibuti | SP7 | 1.14 | 417.25 | 543.78 |
| Dry | Sibuti | SP8 | 0.98 | 20.50 | 19.52 |
| Dry | Sibuti | SP9 | 1.27 | 550.00 | 882.21 |
| Dry | Sibuti | SP10 | 1.50 | 707.43 | 1595.70 |
| Dry | Niah | SP11 | 1.21 | 10.25 | 14.90 |
| Dry | Niah | SP12 | 1.40 | 5.00 | 9.77 |
| Wet | Baram | SP1 | 1.21 | 411.00 | 597.44 |
| Wet | Baram | SP2 | 1.31 | 684.08 | 1165.86 |
| Wet | Baram | SP3 | 1.38 | 549.11 | 1045.93 |
| Wet | Miri | SP4 | 1.47 | 553.17 | 1192.29 |
| Wet | Miri | SP5 | 1.94 | 10.17 | 38.22 |
| Wet | Miri | SP6 | 2.04 | 546.67 | 2274.35 |
| Wet | Sibuti | SP7 | 1.61 | 657.60 | 1714.05 |
| Wet | Sibuti | SP8 | 1.32 | n.d***** | n.d***** |
| Wet | Sibuti | SP9 | 1.43 | 4.25 | 8.73 |
| Wet | Sibuti | SP10 | 1.36 | 654.40 | 1213.67 |
| Wet | Niah | SP11 | 1.43 | 545.00 | 1120.05 |
| Wet | Niah | SP12 | 1.63 | 4.33 | 11.51 |

*****Not determined due to unavailable polymer composition data required for risk calculation.
